# The Host Tissue Repair Vulnerability Index (HTRVI): A Composite Biomarker for Predicting Osteoradionecrosis in Locally Advanced Nasopharyngeal Carcinoma

**DOI:** 10.3390/medsci14040427

**Published:** 2026-07-24

**Authors:** Efsun Somay, Erkan Topkan, Sibel Bascil, Ugur Selek

**Affiliations:** 1 Department of Oral and Maxillofacial Surgery, Faculty of Dentistry, Başkent University, Ankara 06579, Türkiye; 2 Department of Radiation Oncology, Faculty of Medicine, Başkent University, Adana 01120, Türkiye; docdretopkan@gmail.com; 3 Department of Periodontology, Faculty of Dentistry, Başkent University, Ankara 06579, Türkiye; bascil5@yahoo.com; 4 Department of Radiation Oncology, School of Medicine, Koç University, Istanbul 33405, Türkiye; ugurselek@yahoo.com

**Keywords:** osteoradionecrosis, nasopharyngeal carcinoma, host tissue repair vulnerability index, radiotherapy toxicity, host susceptibility, risk stratification

## Abstract

Background: Osteoradionecrosis of the jaw (ORNJ) remains a major late complication of head and neck radiotherapy, and risk assessment relies mainly on clinical and dosimetric factors. We constructed the Host Tissue Repair Vulnerability Index (HTRVI), a composite biomarker integrating inflammatory, immune-nutritional, and oxygenation-related parameters, to estimate biological susceptibility to ORNJ in locally advanced nasopharyngeal carcinoma (LA-NPC). Methods: This retrospective cohort included 261 LA-NPC patients treated with definitive concurrent chemoradiotherapy between 2010 and 2021. HTRVI was calculated as (CRP × platelet × neutrophil)/(albumin × lymphocyte × hemoglobin). HTRVI was compared with hemoglobin (Hb) and the Global Immune-Nutrition-Inflammation Index (GINI) using receiver operating characteristic analysis. Multivariable logistic regression, restricted cubic spline analysis (RCS), and bootstrap resampling assessed independent association, continuous risk relationship, and internal validation. Results: During a median follow-up of 63.8 months, 24 patients (9.2%) developed ORNJ. HTRVI showed superior discrimination (AUC, 0.864; 95% CI, 0.769–0.932) compared with Hb (AUC, 0.785; *p* = 0.001) and GINI (AUC, 0.759; *p* = 0.045). HTRVI remained independently associated with ORNJ after adjustment for mandibular mean dose and post-CCRT tooth extraction burden (OR per standard deviation increase, 4.81; 95% CI, 1.10–20.99; *p* = 0.037). RCS analysis showed a significant continuous association between HTRVI and ORNJ risk (*P*_overall_ < 0.001) without nonlinearity (*P*_nonlinear_ = 0.736). Internal validation showed minimal optimism (bootstrap-corrected AUC, 0.993). Conclusions: HTRVI was independently associated with ORNJ and provided additional predictive information beyond established clinical and dosimetric factors in this single-center cohort. The continuous HTRVI–ORNJ association supports a biological continuum model of host tissue repair vulnerability. However, HTRVI should currently be regarded as an investigational biomarker requiring independent external and prospective validation before clinical implementation.

## 1. Introduction

Osteoradionecrosis of the jaw (ORNJ) remains one of the most debilitating late complications of radiotherapy for head and neck cancer, with reported incidence rates generally ranging from approximately 5% to 10–15% depending on tumor site, treatment technique, mandibular dose exposure, dental status, invasive post-radiotherapy dental procedures, and follow-up duration [1,2,3,4,5]. Importantly, ORNJ has not been eliminated by highly conformal radiation techniques; in a recent proton therapy cohort, Singh et al. reported an ORNJ prevalence of 10.6%, underscoring that this complication remains clinically relevant even in the era of advanced particle therapy [6]. This persistent risk is particularly consequential in locally advanced nasopharyngeal carcinoma (LA-NPC), where excellent locoregional response and favorable long-term survival after concurrent chemoradiotherapy (CCRT), with or without induction or adjuvant systemic therapy, have transformed late radiation morbidity into a major survivorship concern [7,8,9]. For these long-term survivors, ORNJ may impose a prolonged burden through chronic pain, recurrent infection, impaired mastication, dysphagia, speech dysfunction, nutritional compromise, and reduced quality of life, while established disease often requires extended multidisciplinary management ranging from conservative medical care to extensive surgical resection and reconstruction [1,2,3,4,5]. Therefore, accurate identification of patients at increased ORNJ risk before irreversible tissue injury occurs remains a major priority in contemporary radiation oncology and supportive cancer care.

LA-NPC represents a particularly informative clinical setting for investigating ORNJ susceptibility. Unlike many oral cavity and oropharyngeal cancers, where large portions of the mandible may receive relatively uniform high-dose exposure because of direct anatomical proximity to the primary tumor or elective target volumes, LA-NPC treatment typically creates broader mandibular dose gradients across different mandibular subsites. This dosimetric heterogeneity provides an appropriate framework for examining whether ORNJ risk is determined by radiation dose alone or by the interaction between dose exposure and additional clinical or biological risk factors. Moreover, LA-NPC is commonly managed with relatively standardized definitive radiotherapy-based protocols, usually consisting of intensity-modulated radiotherapy combined with concurrent cisplatin, with induction or adjuvant chemotherapy incorporated based on disease burden and institutional practice [7,8,9,10]. Together with the favorable tumor responsiveness and long-term survival achieved in contemporary LA-NPC, this creates a clinically relevant survivorship model in which radiation injury, mandibular dose distribution, dental interventions, and host-related tissue repair vulnerability can be evaluated within the same disease framework [7,8,9,10,11].

Although mandibular radiation exposure remains the most consistently established determinant of ORNJ, dose alone does not fully account for the marked variability observed across clinical series or among individual patients. Even in the modern radiotherapy era, reported ORNJ rates vary substantially, with some cohorts describing very low or near-zero incidence and others reporting rates approaching 10–15%, despite broadly comparable treatment intent and advances in conformal planning [5,10,11,12,13,14,15,16]. Moreover, established non-dosimetric contributors—including baseline dental status, smoking history, periodontal disease, oral infection, chemotherapy exposure, and invasive dental procedures before or after radiotherapy—further indicate that ORNJ risk is shaped by more than mandibular dose distribution alone [5,12,13,14,15,16,17]. These observations suggest that ORNJ results from interactions among radiation-induced tissue injury, local oral and dental vulnerability, treatment-related stressors, and the host’s intrinsic capacity to maintain vascular integrity, regulate inflammation, preserve oxygenation, and repair irradiated bone and soft tissue.

Contemporary evidence indicates that ORNJ develops through interacting biological processes extending beyond direct radiation-induced bone injury. Current pathophysiological models implicate endothelial dysfunction, microvascular rarefaction, chronic inflammation, oxidative stress, fibroatrophic remodeling, impaired bone turnover, and defective tissue regeneration as key contributors to disease initiation and progression [18,19,20,21,22,23]. Importantly, many of these mechanisms are critically dependent on the host’s ability to maintain vascular integrity, regulate inflammatory responses, preserve adequate nutritional and metabolic reserve, sustain tissue oxygenation, and support effective repair of irradiated tissues. Consequently, individuals exposed to similar radiation doses may exhibit markedly different capacities for tissue recovery and long-term skeletal preservation. This conceptual framework suggests that ORNJ may be more appropriately viewed not solely as a radiation dose-dependent complication, but rather as a consequence of the interaction between radiation-induced tissue injury and the host’s intrinsic tissue repair vulnerability. Therefore, quantifying this biological susceptibility may represent a critical step toward improving individualized ORNJ risk assessment and advancing biologically informed survivorship care.

Although numerous systemic inflammatory, immune-nutritional, and composite biomarkers have demonstrated prognostic or predictive utility across oncology, their relevance to ORNJ remains conceptually incomplete [24,25,26,27,28,29,30]. Indices such as the neutrophil-to-lymphocyte ratio, Systemic Immune-Inflammation Index, Pan-Immune-Inflammation Value, Glasgow Prognostic Score, GINI, GLUCAR, and CARWL were largely developed to reflect systemic inflammation, immune-nutritional status, cancer prognosis, treatment tolerance, or selected radiation-related outcomes rather than the integrated biological vulnerability of irradiated mandibular tissues [24,25,26,27,28,29,30]. More importantly, many biomarker studies continue to categorize inherently continuous variables using empirical or data-derived cutoff values, despite well-recognized concerns that dichotomization may reduce statistical power, discard prognostic information, introduce threshold instability, and obscure biologically relevant dose–response relationships [31,32,33]. Consequently, there remains a need for an ORNJ-focused composite biomarker that more explicitly captures host tissue repair vulnerability while preserving the continuous nature of biological risk.

Based on these considerations, we hypothesized that susceptibility to ORNJ reflects not only the magnitude and distribution of mandibular radiation exposure, but also the host’s intrinsic capacity to preserve vascular integrity, regulate inflammation, maintain immune competence, sustain nutritional reserve, and support oxygen-dependent tissue repair. To investigate this hypothesis, we constructed the Host Tissue Repair Vulnerability Index (HTRVI), a multidimensional composite biomarker integrating these biological domains. The primary objective of this study was to evaluate the association between HTRVI and ORNJ in patients with LA-NPC treated with definitive CCRT. Secondary aims were to compare the discriminatory performance of HTRVI with Hb and GINI, assess its independent association with ORNJ after adjustment for established clinical, dental, and dosimetric factors, and characterize the continuous relationship between HTRVI and ORNJ risk.

## 2. Materials and Methods

This study was conducted in accordance with the Declaration of Helsinki and was approved by the Institutional Review Board of Başkent University (Approval No: D-KA 20/58, approval date 7 October 2020). Because of the retrospective study design, the requirement for written informed consent was waived.

### 2.1. Study Design and Patient Population

This retrospective observational cohort study was conducted in the Departments of Oral and Maxillofacial Surgery and Radiation Oncology at Başkent University Adana Research and Application Center. Consecutive patients with newly diagnosed LA-NPC who received curative-intent definitive CCRT between January 2010 and December 2021 were identified from the institutional head and neck cancer database. The study was designed and reported in accordance with the Strengthening the Reporting of Observational Studies in Epidemiology (STROBE) recommendations [34], Appendix A.

Eligible patients were ≥18 years of age, had histopathologically confirmed nasopharyngeal carcinoma, and were staged according to the American Joint Committee on Cancer (AJCC) 8th edition TNM classification [35]. Locally advanced disease was defined as AJCC 8th edition T3–4N0–3M0 or T1–4N1–3M0 disease. Additional eligibility requirements included Eastern Cooperative Oncology Group performance status 0–1, standardized pretreatment dental evaluation before oncologic therapy, complete baseline laboratory measurements obtained within seven days before CCRT, available mandibular dosimetric data, and regular post-treatment oral follow-up.

Patients were excluded if they had previous head and neck radiotherapy, recurrent or metastatic disease at diagnosis, prior mandibular surgery, pre-existing ORNJ, active odontogenic infection requiring emergency intervention, systemic conditions or medications likely to substantially affect inflammation, vascular integrity, bone metabolism, tissue oxygenation, or wound healing, blood transfusion within 30 days before CCRT, incomplete clinical, laboratory, dental, radiological, or dosimetric records, or receipt of induction chemotherapy. The latter criterion was applied to preserve treatment homogeneity because induction chemotherapy was not routinely incorporated into the institutional treatment protocol during the study period.

**Figure 1:**
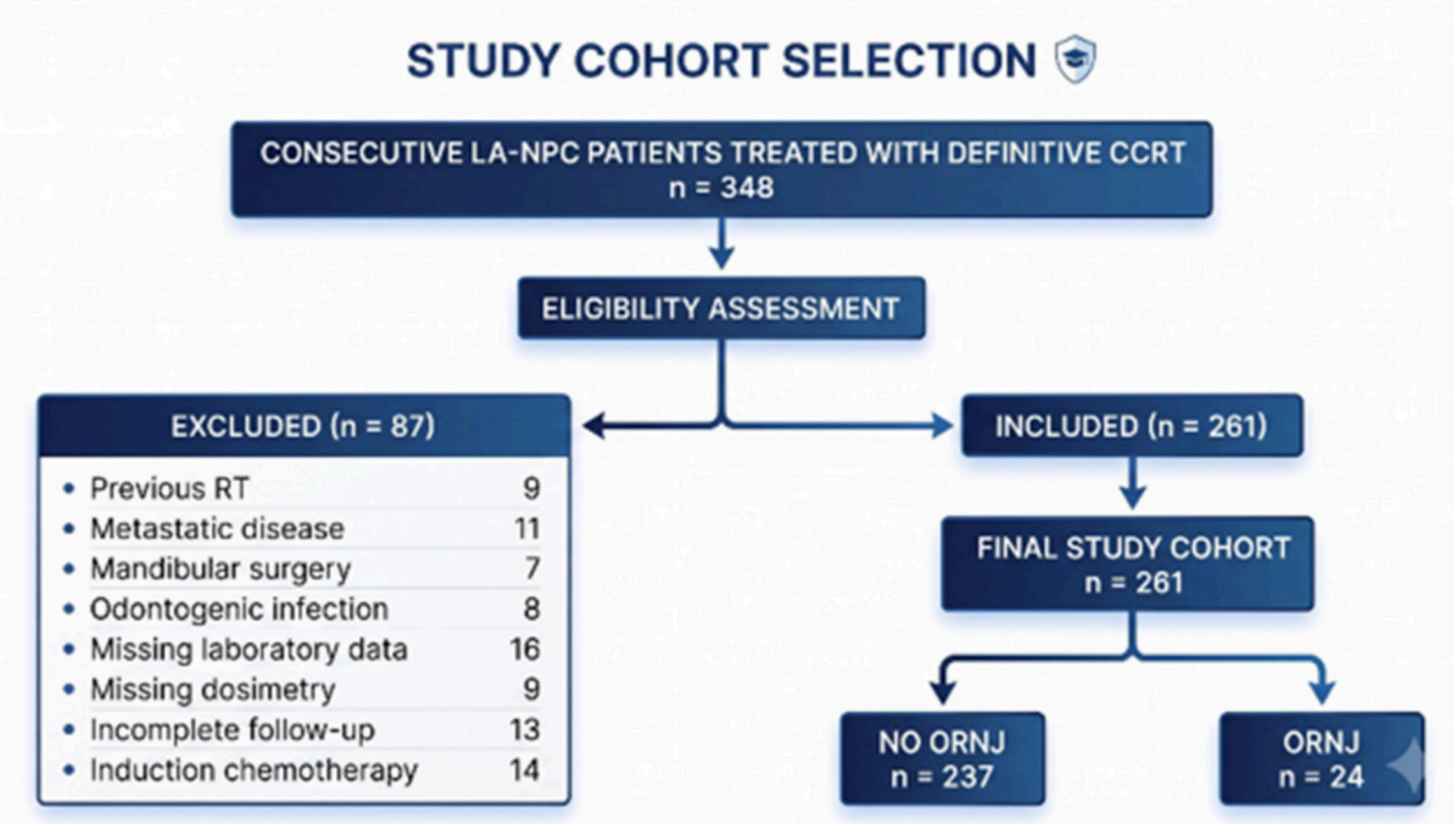
Flowchart of the study cohort selection process.

A flow diagram summarizing patient selection, eligibility assessment, exclusions, and final cohort inclusion is presented in Figure 1.

### 2.2. Pretreatment Dental Evaluation, Oral Management, and Follow-Up

Prior to oncologic treatment, all patients underwent standardized pretreatment dental evaluation by experienced oral and maxillofacial surgeons, in accordance with institutional protocols and established supportive care guidelines for patients undergoing head and neck radiotherapy [5,17]. The evaluation included extraoral and intraoral examinations, periodontal assessment, caries and endodontic assessment, prosthetic and occlusal evaluation, and identification of teeth or restorations with unfavorable long-term prognosis. Panoramic radiographs were routinely obtained, with additional periapical radiographs or cone-beam computed tomography (CBCT) performed when clinically indicated.

Potential sources of oral infection were treated before CCRT whenever feasible. Teeth considered non-restorable because of advanced periodontal disease, extensive structural destruction, severe periapical infection, vertical root fracture, residual roots, or poor long-term prognosis were extracted according to institutional protocols and contemporary recommendations [5]. When extractions were performed, an adequate healing interval was allowed before radiotherapy. Teeth with a favorable prognosis were managed conservatively with periodontal, restorative, endodontic, prosthetic, and preventive measures, including professional cleaning, fluoride application, oral hygiene reinforcement, smoking-cessation counseling when appropriate, and long-term dental surveillance [5].

After completion of CCRT, patients entered a standardized oral surveillance program coordinated by the Departments of Oral and Maxillofacial Surgery and Radiation Oncology. Follow-up visits were scheduled at approximately 1, 3, 6, 9, and 12 months after CCRT and every 6 months thereafter, or more frequently when clinically indicated, in accordance with institutional practice and contemporary recommendations [5]. At each visit, patients were evaluated for mucosal integrity, exposed bone, delayed wound healing, periodontal and dental status, oral hygiene, and signs or symptoms suggestive of ORNJ, including pain, swelling, fistula formation, purulent discharge, pathological tooth mobility, or trismus [5].

Radiographic evaluation was performed when clinical findings suggested possible osseous pathology. Panoramic radiography was used as the first-line imaging modality, whereas periapical radiography, CBCT, or conventional computed tomography was obtained when further characterization of cortical disruption, trabecular destruction, sequestration, pathological fracture, or disease extension was required [2,4,5]. Patients requiring invasive dental procedures after radiotherapy were managed according to institutional preventive protocols aligned with contemporary recommendations for irradiated patients, with conservative treatment prioritized whenever feasible and close monitoring for delayed healing or subsequent ORNJ development [5].

Clinical, radiological, and treatment-related follow-up data were retrospectively reviewed, including the occurrence of ORNJ, the interval from completion of radiotherapy to diagnosis, anatomical location, presumed triggering event, disease stage, therapeutic management, and clinical outcome.

### 2.3. Definition and Severity Assessment of Osteoradionecrosis

During follow-up, ORNJ was clinically diagnosed according to the established conventional definition of exposed irradiated bone, or bone that could be probed through an intraoral or extraoral fistula within the previous radiation field, persisting for at least three months in the absence of persistent, recurrent, or second primary malignancy. For the present analysis, all suspected cases were retrospectively reviewed and adjudicated in accordance with contemporary ISOO–MASCC–ASCO guidance and recent expert recommendations [4,5]. Radiographic evidence of progressive mandibular osteonecrosis supported by compatible clinical findings was also considered during adjudication, particularly in patients without overt bone exposure [4,5].

All suspected ORNJ cases were evaluated by experienced oral and maxillofacial surgeons using clinical examination and radiographic assessment. Panoramic radiography was supplemented by CBCT or conventional CT when required to characterize cortical disruption, trabecular destruction, sequestration, pathological fracture, or disease extension [2,4,17]. When diagnostic uncertainty existed, clinical evolution, radiological findings, and multidisciplinary consensus were used to differentiate ORNJ from recurrent malignancy, osteomyelitis, medication-related osteonecrosis of the jaw, or other mandibular pathologies [4,5]. Disease severity was classified according to the Notani classification [36].

### 2.4. Development of the Host Tissue Repair Vulnerability Index (HTRVI)

The HTRVI was constructed as a composite biomarker to estimate host-related biological susceptibility to ORNJ by integrating routinely available pretreatment laboratory parameters that reflect systemic inflammation, immune competence, nutritional status, and tissue oxygenation. The index was designed to complement established clinical and dosimetric predictors by incorporating host biological characteristics into individualized ORNJ risk assessment.

Pretreatment laboratory parameters were obtained from routine peripheral venous blood samples collected within seven days before initiation of CCRT and before administration of any oncologic treatment. Laboratory measurements included serum C-reactive protein (CRP), albumin, hemoglobin, absolute neutrophil count, lymphocyte count, and platelet count. All analyses were performed in the institutional central laboratory using standardized automated analyzers in accordance with routine clinical practice.

The HTRVI was calculated using the following equation:

$$\mathrm{HTRVI}=\frac{CRP\times \mathrm{platelet count}\times \mathrm{neutrophil count}}{albumin\times \mathrm{lymphocyte count}\times hemoglobin}$$

where CRP was expressed in mg/L; platelet, neutrophil, and lymphocyte counts in ×10^9^/L; and albumin and hemoglobin concentrations in g/dL.

Conceptually, HTRVI integrates four biological domains implicated in radiation-induced tissue injury and repair: systemic inflammation (CRP and neutrophil count), immune competence (lymphocyte count), nutritional reserve (albumin concentration), and tissue oxygenation capacity (hemoglobin concentration). The formulation of HTRVI was based on the hypothesis that host susceptibility to ORNJ reflects the combined effects of interrelated biological processes rather than any single pathway.

The structure of HTRVI was intended to represent the directional balance between biological processes that may impair tissue repair and those that may preserve it. CRP, neutrophil count, and platelet count were placed in the numerator because higher values reflect inflammatory activation, innate immune predominance, and platelet-associated inflammatory and microvascular responses that may accompany impaired healing. In contrast, albumin, lymphocyte count, and hemoglobin were placed in the denominator because higher values indicate greater nutritional reserve, immune competence, and oxygen-carrying capacity. Thus, HTRVI increases when adverse inflammatory features intensify, when protective biological reserves decline, or when both occur simultaneously. This formulation was biologically prespecified rather than selected through data-driven coefficient optimization.

Because the fraction excluding hemoglobin corresponds to the previously described Global Immune–Nutrition–Inflammation Index (GINI), HTRVI may also be expressed as

$$\mathrm{HTRVI}=\frac{GINI}{hemoglobin}$$

To facilitate future reproducibility and potential clinical translation, only routinely available laboratory parameters were incorporated into the index.

### 2.5. Chemoradiotherapy Protocol and Dosimetric Analysis

All patients received definitive CCRT in accordance with institutional treatment protocols and contemporary international guidelines for the management of locally advanced nasopharyngeal carcinoma [7,8,9,10,11]. Target volume delineation was performed using simulation CT fused with contrast-enhanced MRI and, whenever available, 18F-FDG PET/CT. Gross tumor volume (GTV), clinical target volume (CTV), and planning target volume (PTV) were contoured according to international consensus recommendations [8,37].

Intensity-modulated radiotherapy (IMRT) was delivered using a simultaneous integrated boost (SIB) technique. Prescribed doses consisted of 70 Gy to high-risk planning target volumes, 59.4–63 Gy to intermediate-risk planning target volumes, and 54–56 Gy to elective low-risk planning target volumes, delivered in 33 fractions over approximately 6.5 weeks according to institutional protocols and disease extent [9,10,11]. Concurrent chemotherapy consisted of cisplatin 80 mg/m^2^ administered every three weeks during radiotherapy. Following completion of CCRT, eligible patients received adjuvant chemotherapy if they were medically fit and willing to accept treatment, in accordance with multidisciplinary tumor board recommendations and contemporary treatment guidelines [7,8,9,10].

For dosimetric analysis, dose–volume histograms (DVHs) were retrospectively reviewed for all patients. The mandible was contoured according to published recommendations using standardized anatomical definitions [12,13,17]. Extracted dosimetric parameters included mandibular mean dose (Dmean), maximum dose (Dmax), and dose–volume parameters V30, V40, V50, V60, and V70, where Vx represented the percentage of mandibular volume receiving at least Gy. Dosimetric variables were obtained from the original treatment planning system and independently verified before statistical analysis.

Mandibular Dmean was selected a priori as the principal dosimetric covariate because mandibular radiation exposure is the most consistently validated treatment-related determinant of ORNJ and because Dmean provides a stable summary measure of overall mandibular dose burden. Other clinically relevant dose–volume parameters were evaluated descriptively and in exploratory analyses.

### 2.6. Endpoints and Statistical Analysis

The primary endpoint was the occurrence of ORNJ. Secondary analyses compared the discriminatory performance of HTRVI with Hb and GINI, evaluated its independent association with ORNJ, characterized its continuous risk relationship, and internally validated the final model.

Statistical analyses were performed using SPSS version 26.0 (IBM Corp., Armonk, NY, USA) and R version 4.5.1 (R Foundation for Statistical Computing, Vienna, Austria). Continuous variables are presented as mean ± standard deviation or median with interquartile range, as appropriate, and categorical variables as number and percentage. Between-group comparisons used Student’s *t*-test or the Mann–Whitney U test for continuous variables and the chi-square test or Fisher’s exact test for categorical variables.

HTRVI was analyzed primarily as a continuous variable to preserve statistical information and avoid the loss of power, threshold instability, and potential bias associated with dichotomization of continuous predictors [31,32,33]. Discrimination of Hb, GINI, and HTRVI was assessed using receiver operating characteristic curves, with AUCs compared using the DeLong method. Youden-derived cutoffs were reported only for descriptive and comparative purposes.

Associations with ORNJ were evaluated using logistic regression. Because only 24 ORNJ events occurred, multivariable models were restricted to prespecified clinically relevant predictors: mandibular Dmean, post-CCRT tooth-extraction number, and the biomarker of interest. Hb, GINI, and HTRVI were entered in separate models because of their mathematical interdependence. Results are reported as odds ratios with 95% confidence intervals.

The continuous association between HTRVI and ORNJ was examined using restricted cubic spline regression with three degrees of freedom. Internal validation of the final model was performed using 1000 bootstrap resamples, with optimism-corrected AUC, Brier score, and Nagelkerke pseudo-R^2^ reported. All tests were two-sided, with *p* < 0.05 considered statistically significant.

## 3. Results

### 3.1. Patient Characteristics and ORNJ Incidence

A total of 261 patients with LA-NPC who underwent definitive CCRT and met the eligibility criteria were included in the final analysis (Figure 1). During a median follow-up of 63.8 months (range, 5.4–149.6 months), 24 patients (9.2%) developed ORNJ. The median interval from completion of CCRT to ORNJ diagnosis was 13.4 months (range, 7.8–46.7 months). Baseline demographic, clinical, treatment-related, dosimetric, and biological characteristics according to ORNJ status are summarized in Table 1. Compared with patients who did not develop ORNJ, those who developed ORNJ had significantly lower pretreatment Hb concentrations and higher GINI and HTRVI values. They also had significantly higher mandibular Dmean values and a higher number of post-CCRT tooth extractions. In contrast, baseline demographic, disease-related, and treatment-related characteristics, including age, sex, T category, N category, and systemic treatment factors, did not differ significantly between groups (Table 1).

### 3.2. Development and Distribution of HTRVI

**Figure 2:**
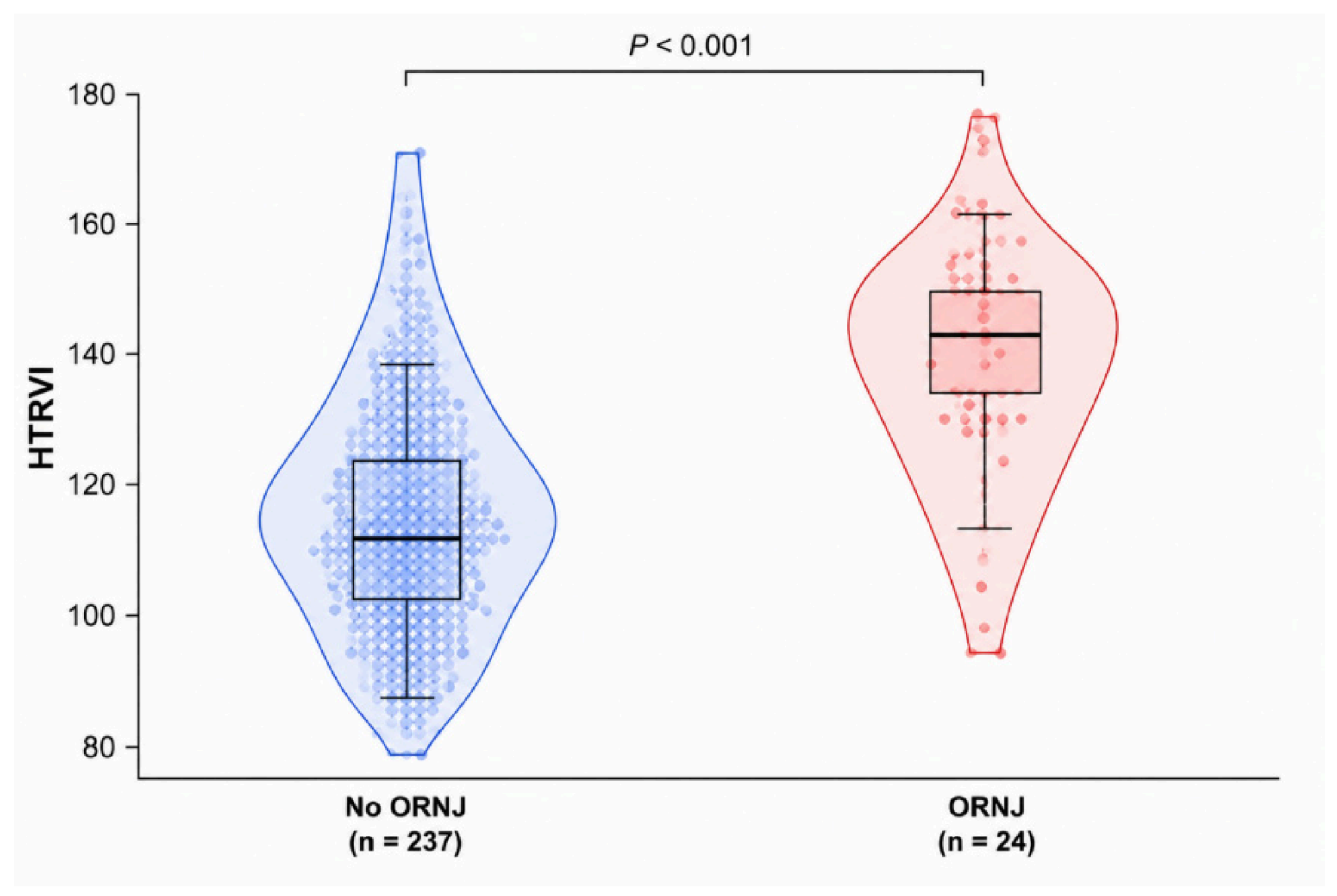
Distribution of the Host Tissue Repair Vulnerability Index (HTRVI) according to osteoradionecrosis of the jaw (ORNJ) status. Violin plots depict the distribution density of HTRVI values, embedded boxplots indicate the median and interquartile range, and individual points represent individual patients. Patients who developed ORNJ had significantly higher HTRVI values than those who did not (median [IQR], 143.3 [131.7–155.1] versus 110.5 [96.7–126.8], respectively; *p* < 0.001, Mann–Whitney U test).

The distribution of HTRVI by ORNJ status is shown in Figure 2. Patients who developed ORNJ had higher HTRVI values than those who did not, with largely distinct distributions and limited overlap. The median HTRVI was 143.3 (IQR, 131.7–155.1) in patients with ORNJ and 110.5 (IQR, 96.7–126.8) in those without ORNJ (*p* < 0.001).

### 3.3. Discriminatory Performance of HTRVI Compared with Hb and GINI

**Figure 3:**
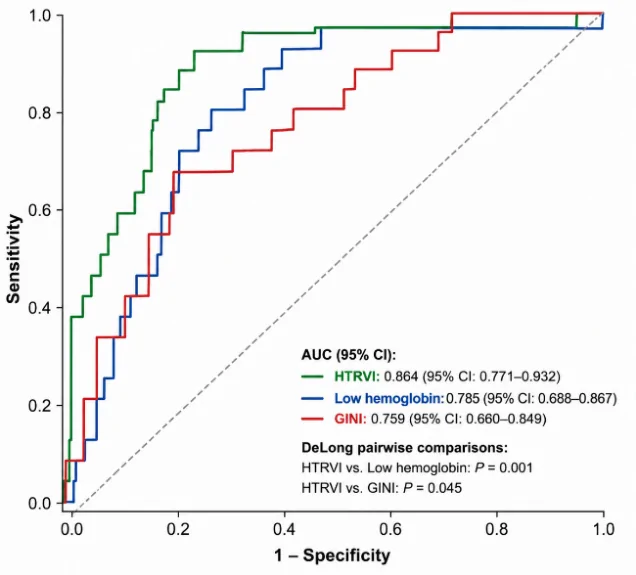
Receiver operating characteristic (ROC) curves comparing the discriminatory performance of the Host Tissue Repair Vulnerability Index (HTRVI), low hemoglobin, and the Global Immune-Nutrition-Inflammation Index (GINI) for predicting osteoradionecrosis of the jaw (ORNJ). Note: HTRVI demonstrated the highest discriminatory performance (AUC, 0.864; 95% CI, 0.769–0.932), followed by low hemoglobin (AUC, 0.785; 95% CI, 0.688–0.867) and GINI (AUC, 0.759; 95% CI, 0.660–0.849). Pairwise comparisons of AUCs were performed using the DeLong method, demonstrating superior discriminatory performance of HTRVI compared with both low hemoglobin (*p* = 0.001) and GINI (*p* = 0.045). Abbreviations: ROC, receiver operating characteristic; AUC, area under the curve; CI, confidence interval; HTRVI, Host Tissue Repair Vulnerability Index; GINI, Global Immune-Nutrition-Inflammation Index; ORNJ, osteoradionecrosis of the jaw. Note: Hemoglobin was modeled as an inverse predictor because lower hemoglobin concentrations were associated with an increased risk of ORNJ; therefore, the ROC curve represents the discriminatory performance of low hemoglobin to maintain a consistent risk orientation across all evaluated biomarkers.

The discriminatory performances of Hb, the GINI, and the HTRVI for predicting ORNJ are summarized in Table 2 and illustrated in Figure 3. HTRVI yielded the highest AUC, at 0.864 (95% CI, 0.769–0.932), compared with low hemoglobin (AUC, 0.785; 95% CI, 0.688–0.867) and GINI (AUC, 0.759; 95% CI, 0.660–0.849). Pairwise comparisons using the DeLong method showed that HTRVI had significantly higher discriminatory performance than both low hemoglobin (*p* = 0.001) and GINI (*p* = 0.045). The optimal HTRVI cutoff identified by the Youden index was 126.8. Corresponding sensitivity, specificity, positive predictive value, negative predictive value, and accuracy estimates for Hb, GINI, and HTRVI are presented in Supplementary Table S1.

### 3.4. Univariable Analyses of ORNJ Risk Factors

Results of the univariable logistic regression analyses are presented in Table 3. Among the evaluated clinical, treatment-related, and host-vulnerability variables, mandibular Dmean, post-CCRT tooth-extraction burden, and HTRVI were significantly associated with the occurrence of ORNJ. In contrast, age, sex, T category, N category, smoking history, and diabetes mellitus were not significantly associated with ORNJ. Hemoglobin and GINI were not included in Table 3 because they constitute the principal biological components of HTRVI and were evaluated separately in the biomarker performance analysis shown in Table 2.

### 3.5. Multivariable Analyses

Multivariable logistic regression analyses are summarized in Table 4. After adjustment for established clinical and treatment-related risk factors, HTRVI remained independently associated with ORNJ. Mandibular mean radiation dose (Dmean) and post-CCRT tooth extraction burden also retained significant independent associations with ORNJ. The final model, incorporating mandibular Dmean, post-CCRT tooth-extraction burden, and HTRVI, demonstrated the most favorable overall performance in predicting ORNJ occurrence.

### 3.6. Continuous Relationship Between HTRVI and ORNJ Risk

**Figure 4:**
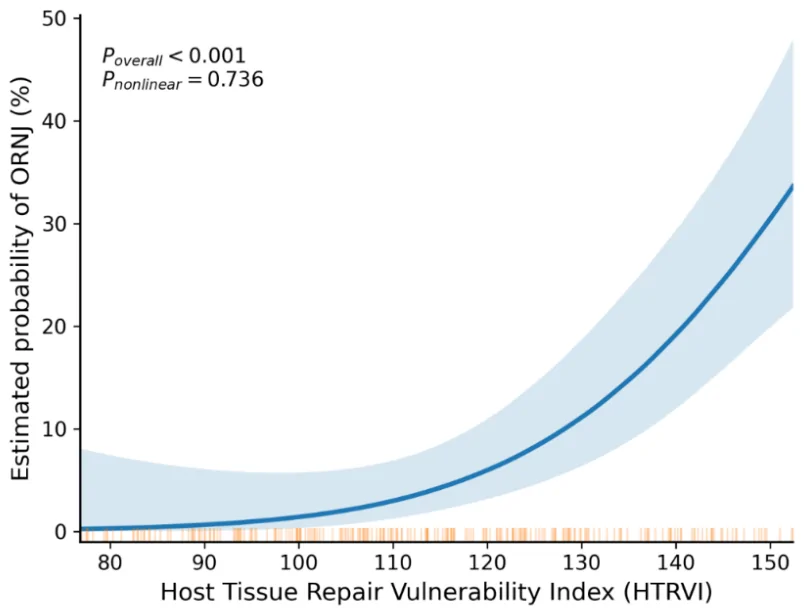
Restricted cubic spline analysis of the association between the Host Tissue Repair Vulnerability Index (HTRVI) and the estimated probability of osteoradionecrosis of the jaw (ORNJ). The solid line represents the fitted probability of ORNJ, and the shaded area indicates the 95% confidence interval. Tick marks along the x-axis denote the distribution of observed HTRVI values. The overall association between HTRVI and ORNJ risk was statistically significant (*p* < 0.001), whereas no significant departure from linearity was observed (*P*_nonlinear_ = 0.736).

The relationship between HTRVI and ORNJ risk was evaluated using RCS analysis (Figure 4). HTRVI demonstrated a significant overall association with ORNJ risk (*p* < 0.001), whereas the test for nonlinearity was not statistically significant (*p* = 0.736). The estimated probability of ORNJ increased progressively with increasing HTRVI values, supporting the treatment of HTRVI as a continuous predictor.

### 3.7. Internal Validation

Internal validation of the final prediction model was conducted using 1000 bootstrap resamples (Supplementary Table S2). The final model demonstrated very high apparent discrimination, which remained high after bootstrap correction. However, because only 24 ORNJ events were available, these estimates remain vulnerable to event-limited instability and should not be interpreted as evidence of externally reproducible predictive performance. The optimism-corrected AUC was 0.993, the corrected Nagelkerke pseudo-R^2^ was 0.759, and the corrected Brier score was 0.022.

## 4. Discussion

This study developed and evaluated HTRVI as a composite biomarker of host-related biological susceptibility to ORNJ in patients with LA-NPC treated with definitive CCRT. HTRVI demonstrated stronger discriminatory performance than its constituent biomarkers, Hb and GINI, and remained independently associated with ORNJ after adjustment for established clinical, dental, and dosimetric risk factors, including mandibular Dmean and post-CCRT tooth extraction burden. RCS analysis showed a continuous association between HTRVI and ORNJ risk without evidence of a nonlinear threshold, consistent with the concept that host tissue repair vulnerability exists along a biological continuum. Addition of HTRVI to the clinical–dosimetric model further improved model performance, suggesting that its integration with treatment-related variables may warrant further evaluation in future multivariable ORNJ risk models.

A key finding of the present research was the continuous association between HTRVI and ORNJ risk. RCS analysis confirmed a statistically significant relationship between HTRVI and ORNJ (*p* < 0.001), without evidence of nonlinearity (*p* = 0.736). These results indicate that ORNJ risk increases progressively with increasing HTRVI values rather than emerging at a distinct biological threshold. Accordingly, HTRVI appears to function as a continuous biomarker of host tissue repair vulnerability rather than a dichotomous risk classifier. Unlike previous ORNJ biomarker studies, all of which have predominantly relied on dichotomized biomarker analyses based on empirically derived cutoff values [28,37,38], the present study evaluated HTRVI primarily as a continuous variable and demonstrated a progressive increase in ORNJ risk across its full observed range. This distinction is methodologically important because categorization of continuous predictors may reduce statistical power, distort dose–response relationships, introduce threshold instability, and limit generalizability across patient populations [32,33,34].

The second principal finding was the superior discriminatory ability of HTRVI compared with its individual components, Hb and the GINI. HTRVI yielded the highest area under the curve (AUC) for ORNJ prediction (0.864; 95% CI, 0.769–0.932), surpassing Hb (AUC, 0.785; 95% CI, 0.686–0.865; DeLong *p* = 0.001) and GINI (AUC, 0.759; 95% CI, 0.658–0.849; DeLong *p* = 0.045). The strength of association with ORNJ was also greatest for HTRVI (odds ratio [OR] per standard deviation increase, 4.45; 95% CI, 2.55–7.77), compared with GINI (OR, 2.60; 95% CI, 1.65–4.11) and Hb (OR, 0.34; 95% CI, 0.19–0.59). These results indicate that combining inflammatory, immune-nutritional, and oxygenation-related parameters yields a more robust measure of host tissue repair vulnerability than any single component alone. This interpretation is supported by the broader oncologic biomarker literature, in which multidomain composite indices may enhance risk stratification compared with single-domain markers [25,26,27,28,29,30,31].

The observed incremental discriminatory value of HTRVI relative to Hb and GINI is biologically plausible because ORNJ arises from several interacting biological impairments rather than from a single isolated pathway. Radiation-induced mandibular injury involves endothelial dysfunction, microvascular compromise, persistent inflammation, oxidative stress, fibroatrophic remodeling, impaired bone turnover, and defective tissue regeneration [18,19,20,21,22,23]. HTRVI integrates markers related to inflammatory activation and innate immune response in its numerator and markers of immune competence, nutritional reserve, and oxygen-carrying capacity in its denominator. These parameters have been implicated in radiation injury, wound healing, and oncologic risk stratification [18,19,20,21,22,23,24,25,26,27,28,29,30]. HTRVI therefore represents the balance between adverse biological stress and the host resources available to sustain tissue repair. Neither Hb nor GINI alone captures this complete biological balance: Hb primarily reflects oxygen-carrying capacity, whereas GINI integrates inflammatory and immune-nutritional status without directly incorporating oxygenation-related reserve.

From a statistical perspective, combining partially complementary biological information may improve discrimination when the constituent variables capture nonidentical dimensions of susceptibility and provide concordant risk information [25,26,27,28,29,30,31]. A patient with modest abnormalities across several domains may have substantial aggregate vulnerability even when no individual marker is markedly abnormal. Conversely, a single biomarker may provide an incomplete or noisy representation of the underlying biological construct. HTRVI may therefore produce a stronger aggregate risk signal by summarizing multiple correlated but biologically nonredundant dimensions within a single continuous measure. Nevertheless, the superior performance of HTRVI in the present cohort should be regarded as an empirical finding requiring external confirmation rather than as an inherent property of composite indices.

Recent developments in precision oncology have highlighted the potential value of multimodal prediction strategies that integrate complementary biological data sources rather than relying on a single biomarker modality. In nasopharyngeal carcinoma, functional imaging biomarkers derived from 18F-FDG PET/CT have demonstrated predictive or prognostic value for treatment response, disease outcomes, and the identification of patients with higher oncologic risk by characterizing tumor-related metabolic activity, burden, and biological heterogeneity [39,40]. These imaging biomarkers, however, primarily interrogate tumor-related characteristics and do not directly assess host factors that may influence susceptibility to radiation-induced normal tissue injury. In contrast, HTRVI was specifically designed to estimate host tissue repair vulnerability by integrating inflammatory burden, immune competence, nutritional reserve, and oxygen-carrying capacity. Accordingly, imaging-derived and host-related biomarkers should be regarded as complementary rather than competing approaches. Future multimodal prediction models integrating tumor-related imaging features with host biological biomarkers may provide a more comprehensive framework for individualized risk stratification in nasopharyngeal carcinoma; however, such approaches require dedicated development and validation for normal tissue toxicity endpoints, including ORNJ, rather than extrapolation from models developed for tumor response or survival.

Importantly, the present findings should not be interpreted as diminishing the established role of treatment-related determinants of ORNJ. Consistent with previous studies, mandibular Dmean and post-CCRT tooth extraction burden remained the strongest treatment-related predictors of ORNJ in the present cohort [2,3,4,5,12,13,14,15,16,17,18]. However, the persistence of HTRVI as an independent predictor after adjustment for mandibular Dmean and post-CCRT tooth extraction burden suggests that host-related biological susceptibility provides complementary predictive information beyond the magnitude of local tissue injury alone. This observation supports a conceptual model in which mandibular Dmean and post-treatment dental trauma contribute to the extent of local tissue insult, whereas the host’s intrinsic capacity for vascular maintenance, inflammatory regulation, tissue oxygenation, and wound healing influences whether irradiated mandibular tissues recover or progress to ORNJ [2,3,4,5,12,13,14,15,16,17,18,19,20,21,22,23].

These findings suggest potential future clinical relevance but should presently be interpreted within an investigational framework. HTRVI is not intended to replace established clinical, dental, or dosimetric predictors of ORNJ, and the current retrospective single-center findings do not support its use for directing individual patient management. If independently and prospectively validated, incorporation of host-related biological vulnerability into existing risk models may contribute to more refined ORNJ risk stratification and risk-adapted supportive-care strategies. Patients identified as having greater host tissue repair vulnerability might undergo more intensive pre- and post-radiotherapy dental assessment, shorter surveillance intervals, earlier management of periodontal or mucosal disease, reinforcement of oral-hygiene measures and nutritional support, and closer monitoring following dental extraction or other mandibular trauma. HTRVI could also be evaluated alongside established clinical and dosimetric variables when considering preventive dental procedures, extraction timing, or mandibular dose-reduction strategies during radiotherapy planning, provided that tumor coverage and oncologic efficacy are not compromised. HTRVI may additionally warrant investigation as a biological component of multivariable normal tissue complication probability models integrating clinical and dosimetric factors [12,13,14,15,16,17,18,25,26,27,28,29,30,31]. However, these potential applications remain hypothetical. At present, neither validated HTRVI risk thresholds nor interventional evidence demonstrating improved ORNJ outcomes with HTRVI-guided management is available. Demonstration of reproducibility, calibration, incremental clinical value, and treatment utility in independent prospective cohorts will therefore be required before routine clinical implementation.

This study has several methodological strengths. The analysis was conducted in a homogeneous cohort of patients with locally advanced nasopharyngeal carcinoma treated with definitive IMRT-based CCRT, reducing heterogeneity related to tumor site and treatment intent. Standardized institutional protocols were used for pretreatment dental assessment, post-treatment oral surveillance, and mandibular dosimetric evaluation, supporting consistency in data collection. HTRVI was analyzed primarily as a continuous variable, and its association with ORNJ was further examined using RCS analysis to assess potential nonlinearity. The final prediction model underwent internal validation using bootstrap resampling, consistent with contemporary recommendations for assessing prediction models. These features strengthen the internal validity and methodological rigor of the present analysis.

Several limitations of this study should be acknowledged in interpreting the findings. First, the retrospective single-center design introduces the possibility of selection bias and residual confounding, despite the inclusion of consecutive patients and standardized protocols. Second, only 24 patients developed ORNJ, which limited the effective information available for multivariable model development and necessitated a deliberately parsimonious modeling strategy. Although the number of predictors was restricted a priori and internal validation was performed using bootstrap resampling, prediction models based on relatively few outcome events remain susceptible to coefficient instability, model optimism, and exaggerated performance estimates. The remarkably high discrimination of the final model should therefore be interpreted cautiously. In particular, the AUC may partly reflect the strong separation produced by mandibular Dmean and post-CCRT tooth-extraction burden within this cohort rather than performance that would necessarily be reproduced in an independent population. Bootstrap correction quantifies internal optimism but cannot overcome the uncertainty associated with the limited number of outcome events or establish external validity. Accordingly, the reported performance metrics should be considered preliminary and require confirmation in substantially larger external cohorts with an adequate number of ORNJ events. Third, HTRVI was derived from a single pretreatment blood sample, which does not capture temporal changes in the relevant biological domains during and after CCRT. Fourth, HTRVI was constructed using routinely available laboratory parameters and does not incorporate molecular biomarkers, cytokine profiles, genomic susceptibility markers, radiomic features, or other biological determinants that may further improve individualized risk prediction. Finally, HTRVI was developed and internally validated within the same cohort, and bootstrap validation cannot establish transportability to independent populations. Accordingly, the reported predictive performance should be interpreted cautiously and should not be interpreted as evidence supporting routine clinical implementation. External validation in independent LA-NPC cohorts, followed by prospective evaluation in independent populations treated under different clinical, radiotherapy, dental-management, and supportive-care protocols, is required to establish the reproducibility, calibration, generalizability, and potential clinical utility of HTRVI.

## 5. Conclusions

In this single-center retrospective cohort of patients with LA-NPC, HTRVI was independently associated with ORNJ and provided complementary predictive information beyond mandibular radiation dose and post-CCRT tooth-extraction burden. Its continuous association with ORNJ supports the concept that host tissue repair vulnerability exists along a biological continuum rather than at a discrete threshold. Nevertheless, HTRVI remains an investigational biomarker, and the present findings should be regarded as hypothesis-generating. Independent external and prospective validation is required to confirm its reproducibility, calibration, generalizability, and clinical utility before incorporation into routine ORNJ risk assessment or treatment decision-making.

## Figures and Tables

**Table 1:** Patient, treatment, oral, and biomarker characteristics according to ORNJ status.

Variable	Total (n = 261)	No ORNJ (n = 237)	ORNJ (n = 24)	*p*
Age, years	56.0 (47.0–64.0)	56.0 (47.0–64.0)	54.5 (44.8–66.0)	0.882
Male sex, n (%)	171 (65.5)	156 (65.8)	15 (62.5)	0.822
ECOG 1, n (%)	139 (53.3)	126 (53.1)	13 (54.1)	0.798
Smoking, n (%)	222 (85.1)	201 (84.8)	21 (87.5)	0.902
Alcohol use, n (%)	141 (54.0)	128 (54.0)	13 (54.2)	0.914
Diabetes mellitus, n (%)	41 (15.7)	37 (15.6)	4 (16.7)	0.777
Cardiovascular disease, n (%)	77 (29.5)	72 (30.4)	5 (20.8)	0.481
Undifferentiated histology, n (%)	229 (87.7)	208 (87.7)	21 (87.5)	0.826
Post-CCRT tooth extraction number	1.0 (1.0–2.0)	1.0 (1.0–2.0)	3.5 (2.0–4.0)	<0.001
Mandibular Dmean, Gy	34.6 (27.9–40.3)	32.9 (27.1–38.4)	55.8 (52.0–60.2)	<0.001
Hemoglobin, g/dL	12.1 (10.7–14.0)	12.4 (10.8–14.1)	10.5 (9.7–10.8)	<0.001
GINI	1374 (1284–1444)	1373 (1283–1427)	1502 (1390–1556)	<0.001
HTRVI	113.7 (98.1–130.0)	110.5 (96.7–126.8)	143.3 (131.7–155.1)	<0.001

Abbreviations: ORNJ, osteoradionecrosis of the jaw. Note: Data are presented as median (interquartile range) or number (%), as appropriate. Values are median (IQR) or n (%). Continuous variables were compared using the Mann–Whitney U test and categorical variables using the χ^2^ test or Fisher’s exact test, as appropriate.

**Table 2:** Comparative discriminatory performance of Hb, GINI, and HTRVI for osteoradionecrosis prediction.

Variable	No ORNJ Median (IQR)	ORNJ Median (IQR)	OR per SD (95% CI)	*p*	AUC (95% CI)
Hb	12.4 (10.8–14.1)	10.5 (9.7–10.8)	0.34 (0.19–0.59)	<0.001	0.785 (0.686–0.865)
GINI	1373 (1283–1427)	1503 (1390–1556)	2.60 (1.65–4.11)	<0.001	0.759 (0.658–0.849)
HTRVI	110.5 (96.7–126.8)	143.3 (131.7–155.1)	4.45 (2.55–7.77)	<0.001	0.864 (0.769–0.932)

Abbreviations: ORNJ, osteoradionecrosis of the jaw; Hb, hemoglobin; GINI, Global Immune-Nutrition-Inflammation Index; HTRVI, Host Tissue Repair Vulnerability Index; OR, odds ratio; SD, standard deviation; AUC, area under the receiver operating characteristic curve; IQR, interquartile range. Note: Odds ratios represent the change in ORNJ risk per one standard deviation increase in the respective biomarker. Pairwise AUC comparisons were performed using the DeLong method. HTRVI demonstrated superior discriminatory performance compared with Hb (ΔAUC = 0.079, *p* = 0.001) and GINI (ΔAUC = 0.104, *p* = 0.045).

**Table 3:** Univariable logistic regression analysis of clinical, treatment-related, and host tissue repair vulnerability factors associated with osteoradionecrosis of the jaw.

Variable	Unit/Comparison	OR (95% CI)	*p* Value
Age	Per 10-year increase	0.95 (0.7–1.31)	0.752
Male	vs. female	0.87 (0.36–2.06)	0.744
T3–4	vs. T1–2	1.17 (0.87–1.39)	0.260
N2–3	vs. N0–1	1.09 (0.82–1.36)	0.571
Smoking history	Yes vs. no	1.25 (0.36–4.42)	0.725
Diabetes mellitus	Yes vs. no	1.08 (0.35–3.34)	0.892
Post-CCRT tooth extraction number	Per additional tooth	3.09 (2.12–4.50)	<0.001
Mandibular Dmean	Per 5-Gy increase in mandibular Dmean	8.12 (3.48–18.95)	<0.001
HTRVI	Per SD increase	4.45 (2.55–7.77)	<0.001

Abbreviations: OR, odds ratio; CI, confidence interval; CCRT, concurrent chemoradiotherapy; Dmean, mean mandibular radiation dose; HTRVI, Host Tissue Repair Vulnerability Index. Note: Odds ratios for continuous variables are reported per specified unit or per one standard deviation, as indicated. Hb and GINI were evaluated separately because of their mathematical relationship with HTRVI; their comparative performance is presented in Table 2.

**Table 4:** Multivariable logistic regression models for ORNJ prediction.

Variable	Model 1: Clinical-Dosimetric	Model 2: +Hb	Model 3: +GINI	Model 4: +HTRVI
Mandibular Dmean, per 5-Gy increase	9.55 (3.24–28.11), *p* < 0.001	9.29 (2.98–29.02), *p* < 0.001	9.32 (2.88–30.19), *p* < 0.001	9.25 (2.49–34.31), *p* < 0.001
Post-CCRT extraction number, per tooth	3.63 (1.48–8.87), *p* = 0.005	3.17 (1.29–7.82), *p* = 0.012	3.46 (1.31–9.09), *p* = 0.012	3.19 (1.20–8.49), *p* = 0.020
Hemoglobin, per SD increase	—	0.28 (0.07–1.12), *p* = 0.072	—	—
GINI, per SD increase	—	—	2.95 (1.00–8.72), *p* = 0.051	—
HTRVI, per SD increase	—	—	—	4.81 (1.10–20.99), *p* = 0.037
AIC	37.37	35.39	34.72	32.15
AUC	0.994	0.995	0.996	0.995
LR test vs. Model 1	Reference	χ^2^ = 3.98, *p* = 0.046	χ^2^ = 4.65, *p* = 0.031	χ^2^ = 7.23, *p* = 0.007

Abbreviations: ORNJ, osteoradionecrosis of the jaw; CCRT, concurrent chemoradiotherapy; Dmean, mean mandibular radiation dose; Hb, hemoglobin; GINI, Global Immune-Nutrition-Inflammation Index; HTRVI, Host Tissue Repair Vulnerability Index; SD, standard deviation; AIC, Akaike information criterion; AUC, area under the receiver operating characteristic curve; LR, likelihood ratio. Note: Values are ORs (95% CIs) unless otherwise indicated. Hb, GINI, and HTRVI were evaluated in separate models because of mathematical interdependence. Lower AIC indicates better model fit.

## Data Availability

The original contributions presented in this study are included in the article/Supplementary Material. Further inquiries can be directed to the corresponding author.

## Supplementary Materials

The following supporting information can be downloaded at https://www.mdpi.com/article/10.3390/medsci14040427/s1, Table S1: Diagnostic performance characteristics of Hb, GINI, and HTRVI for predicting osteoradionecrosis of the jaw; Table S2: Apparent and bootstrap-corrected performance of the final HTRVI prediction model for ORNJ.

## Appendix A. STROBE Guidelines

**Section**	**Item No.**	**STROBE Recommendation**	**Reported in Manuscript**
Title and Abstract	1a	Indicate the study design using a commonly used term in the title or abstract.	Abstract (Recommend adding “Retrospective Cohort Study” to the title or abstract.)
	1b	Provide an informative and balanced abstract summarizing the study.	Abstract
Introduction	2	Explain the scientific background and rationale.	Pages 1–4
	3	State the objectives and prespecified hypotheses.	Page 4
Methods	4	Present key elements of study design early in the paper.	Page 5
	5	Describe the setting, locations, and relevant dates.	Page 5
	6a	Give the eligibility criteria and describe participant selection.	Pages 5–6
	6b	Describe matching criteria (if applicable).	Not applicable
	7	Clearly define outcomes, exposures, predictors, confounders, and effect modifiers.	Pages 9–13
	8	Describe data sources and methods of assessment.	Pages 10–12
	9	Describe efforts to address potential sources of bias.	Pages 5–6 (could be expanded)
	10	Explain how the study size was determined.	Consecutive cohort; recommend explicitly stating no formal sample size calculation
	11	Explain handling of quantitative variables.	Pages 10–14
	12a	Describe all statistical methods, including confounding control.	Pages 12–14
	12b	Describe methods for subgroup or interaction analyses.	Not applicable
	12c	Explain how missing data were addressed.	Complete-case analysis implied; recommend explicit statement
	12d	Explain how loss to follow-up was addressed.	Patients with follow-up <12 months excluded (Page 5)
	12e	Describe sensitivity analyses.	Bootstrap validation (Page 20)
Results	13a	Report the number of individuals at each stage of the study.	Figure 1; Pages 5–6
	13b	Give reasons for non-participation at each stage.	Figure 1
	13c	Consider use of a flow diagram.	Figure 1
	14a	Describe participant characteristics.	Table 1
	14b	Indicate the number of participants with missing data.	None (recommend explicitly stating complete-case analysis)
	14c	Summarize follow-up time.	Page 14
	15	Report numbers of outcome events.	Pages 14–15
	16a	Present unadjusted and adjusted estimates with confidence intervals.	Table 3 and Table 4
	16b	Report category boundaries when continuous variables are categorized.	Supplementary Table S1
	16c	Translate relative risk estimates into absolute risk where appropriate.	Not applicable
	17	Report additional analyses (subgroups, interactions, sensitivity analyses).	ROC, spline analysis, bootstrap validation (Pages 16–20)
Discussion	18	Summarize key findings with reference to study objectives.	Pages 22–31
	19	Discuss study limitations and potential bias.	Pages 29–30
	20	Provide an overall interpretation considering objectives and previous evidence.	Pages 22–31
	21	Discuss the generalizability of the findings.	Pages 29–30
Other Information	22	State the source of funding and the funders’ roles.	To be added if applicable

## Appendix B. REMARK Checklist

**Section**	**Item**	**REMARK Recommendation**	**Reported in Manuscript**
Introduction	1	State the prespecified study objectives and hypotheses.	Page 4
Materials and Methods	2	Describe patient characteristics, eligibility criteria, patient source, treatments received, and the time period.	Pages 5–12
	3	Describe patient selection, inclusion and exclusion criteria, and follow-up.	Pages 5–9
	4	Clearly define the clinical endpoint(s).	Pages 9–10
	5	Describe candidate biomarkers and rationale for their selection.	Pages 10–11
	6	Describe specimen characteristics, timing of collection, and storage conditions.	Pretreatment blood sampling is described (Page 10); storage/pre-analytical handling should be added
	7	Describe assay methods, laboratory procedures, quality control, and reproducibility.	Routine laboratory methods described (Page 10); manufacturer/analyzer information recommended
	8	State whether laboratory personnel were blinded to outcomes.	Not reported
	9	Explain how biomarker values were handled in statistical analyses.	Pages 10–14
	10	Describe study size and rationale.	Consecutive cohort; explicit statement recommended
	11	Describe statistical methods, variable selection, and model-building strategy.	Pages 12–14
	12	Explain handling of missing data.	Complete-case analysis implied; explicit statement recommended
	13	Describe internal or external validation methods.	Bootstrap internal validation (Page 20)
Results	14	Describe participant flow.	Figure 1
	15	Report patient characteristics and number of outcome events.	Table 1
	16	Report distribution of biomarker values.	Figure 2
	17	Report associations between biomarker and outcome.	Table 2, Table 3 and Table 4
	18	Present multivariable analyses including established prognostic factors.	Table 4
	19	Report model performance measures (AUC, calibration, discrimination).	ROC, AUC, bootstrap validation reported; calibration plot not included
	20	Report validation results.	Bootstrap validation (Page 20)
Discussion	21	Interpret the findings in light of the study objectives and prior evidence.	Pages 22–31
	22	Discuss study limitations.	Pages 29–30
	23	Discuss clinical implications and future validation.	Pages 30–31
