# Supplementary material for: The Host Tissue Repair Vulnerability Index (HTRVI): A Composite Biomarker for Predicting Osteoradionecrosis in Locally Advanced Nasopharyngeal Carcinoma
Source: Med Sci (Basel). 2026 Jul 24;14(4):427. doi: 10.3390/medsci14040427 (PMC13515248; doi:10.3390/medsci14040427)
Supplement: Supplementary file 1 [file medsci-14-00427-s001.zip › medsci-4460218-supplementary.pdf]

Supplementary Tables

Supplementary Table S1. Diagnostic performance characteristics of Hb, GINI, and HTRVI for predicting osteoradionecrosis of the jaw

Biomarker	Optimal cutoff	Sensitivity (%)	Specificity (%)	PPV (%)	NPV (%)	Youden index	Accuracy (%)
Low Hb	≤11.4 g/dL	79.2	72.6	23.5	97.1	0.518	73.2
GINI	>1457	83.3	69.2	22.7	97.5	0.525	70.5
HTRVI	>126.8	87.5	80.6	31.3	98.4	0.681	81.2

Abbreviations: Hb, hemoglobin; GINI, Global Immune-Nutrition-Inflammation Index; HTRVI, Host Tissue Repair Vulnerability Index; PPV, positive predictive value; NPV, negative predictive value.

Note: Optimal cutoff values were determined using the Youden index derived from receiver operating characteristic (ROC) analysis. Positive predictive value (PPV) and negative predictive value (NPV) were calculated using the observed prevalence of osteoradionecrosis in the study cohort (24/261, 9.2%).

Supplementary Table S2. Apparent and bootstrap-corrected performance of the final HTRVI prediction model for ORNJ.

Performance metric	Apparent estimate	Bootstrap optimism	Bootstrap-corrected estimate
Area under the ROC curve (AUC)	0.995	0.002	0.993
Brier score	0.016	0.006	0.022
Nagelkerke R^2	0.886	0.127	0.759
Akaike information criterion (AIC)	32.15	—	—
Bayesian information criterion (BIC)	46.40	—	—

Abbreviations: AUC, area under the receiver operating characteristic curve; AIC, Akaike information criterion; BIC, Bayesian information criterion; HTRVI, Host Tissue Repair Vulnerability Index; ORNJ, osteoradionecrosis of the jaw. **Note:** Internal validation was performed using 1000 bootstrap resamples. Bootstrap optimism was estimated as the mean difference between apparent and test performance across bootstrap samples. The final prediction model included mandibular Dmean, the number of teeth extracted after CCRT, and the Host Tissue Repair Vulnerability Index (HTRVI). The calibration intercept and slope were not reported because near-complete separation resulted in unstable bootstrap calibration estimates.
